# Comparative Composition, Interfacial Properties, and Antioxidant Activity of Flaxseed Protein Isolates from Different Varieties

**DOI:** 10.3390/foods15101808

**Published:** 2026-05-20

**Authors:** Xiao Yu, Chen Zhang, Haohe Sun, Yingying Zhu, Dengfeng Peng, Qianchun Deng, Lili Zhang, Limin Wang

**Affiliations:** 1 Key Laboratory of Cold Chain Food Processing and Safety Control, Ministry of Education, College of Food and Bioengineering, Zhengzhou University of Light Industry, Zhengzhou 450001, China; zhangchen011121@163.com (C.Z.); yuzigao1204@163.com (H.S.); zhuying881020@163.com (Y.Z.); 2 Hubei Key Laboratory of Lipid Chemistry and Nutrition, Key Laboratory of Oilseeds Processing, Ministry of Agriculture, Oil Crops Research Institute, Chinese Academy of Agricultural Sciences, Wuhan 430062, China; dfpengfood@outlook.com; 3 Zhangjiakou Academy of Agricultural Sciences, Zhangjiakou 075000, China; zhanglili57@126.com; 4 Crop Research Institute, Gansu Academy of Agricultural Sciences, Lanzhou 730070, China; wanglm@gsagr.cn

**Keywords:** flaxseed protein isolate, flaxseed varieties, composition structure, interfacial properties, antioxidant activities, phenolic compounds

## Abstract

The present study aimed to compare the composition structure, interfacial, and antioxidant activities of flaxseed protein isolates (FPIs) in different flaxseed varieties. The results showed that apparently intact protein bodies (PBs) were manifested as densely staining cytoplasmic inclusions with distinct boundaries and varying diameter ranges among different flaxseed varieties. Through alkali extraction with isoelectric precipitation, FPIs exhibited a relatively small and irregular lamellar strip structure with varying sizes and shapes packed with spherical particles in studied flaxseed varieties. The different composition structures of FPIs among studied flaxseed varieties were also obtained, involving the protein subunits’ intrinsic fluorescence properties, secondary structures, and amino acid profiles. These structural differences also led to differential purities, aqueous solubility, dispersion properties, and surface charges. Moreover, the varying emulsifying and foaming properties of FPIs from different flaxseed varieties were also observed due to the formation of coarse lipid droplets (5~40 μm) and foams (20~100 μm) with the specific structure of the oil/gas–water interface and bulk aqueous phase. The retention of phenolic compounds into FPIs still displayed evident variety specificity from 323 to 478 mg/100 g and 210 to 347 mg/100 g, which definitely led to escalated antioxidant activities. Thus, FPIs from Longya 13# and Neiya 9# flaxseed varieties were screened for favorable emulsifying and foaming properties due to the balanced molecular rigidity/unfolding and interfacial adsorption/stabilization behavior.

## 1. Introduction

Flaxseed is commonly acknowledged as a superfood that provides potential health benefits owing to its abundant content of essential fatty acid (α-linolenic acid, ALA), high-quality protein, soluble dietary fiber, and bioactive compounds (lignan, etc.) [1]. Flaxseed is commercially available for consumption as whole seed, partially defatted flours, sauce, milk, etc., due to its superior complex of multiple nutrients. Moreover, flaxseed is also used to obtain proteins, gum polysaccharides, and lignan extracts from defatted flours for application in the healthy food system [2]. According to data from FAOSTAT, global flaxseed production reached almost 3.07 million metric tons in 2022, with China accounting for 0.34 million metric tons. Total flaxseed imports amounted to 0.43 million metric tons, with Russia supplying 0.30 million metric tons—representing 70% of the total imports. Accordingly, generating substantial quantities of defatted flaxseed flours with abundant proteins should result in its efficient and high-value utilization in healthy food instead of its current application in animal feed or fertilizer.

Flaxseed, as the most widely cultivated economic oil crop in China, has been located mainly in the northwest and northern regions of China, including Gansu, Ningxia, Xinjiang, Inner Mongolia, Shanxi, Hebei, etc. Currently, there are 30~40 flaxseed varieties grown in China, including in Tianya, Longya, Ningya, Jinya, Baya, etc., to achieve short fertility, a rich yield, and high oil or protein contents, as well as desirable resistance to drought, collapse, blight and wilt disease, etc. As previously reported, different flaxseed varieties were classified by applying a multivariate analysis based on the abundance of main nutrients, bioactive concomitants, and antioxidant activities to optimize processing for the utilization of whole seed or extraction of specific bioactive components [3]. Then, commercially available flaxseed varieties in Egypt with the highest nutritional and medicinal attributes but lower levels of cyanogenic glycosides were screened using a comprehensive metabolomic approach [4]. Moreover, species-specific flaxseed proteins and peptides were obtained to distinguish flaxseed varieties by using proteomic analysis [5]. However, comparative studies of the techno-functionality of proteins obtained from different flaxseed varieties are relatively limited, which is pivotal for their application in healthy food.

Flaxseed proteins were consistently accumulated in the protein storage vacuoles (PSVs) of endosperm cells during seed maturation, which subsequently produced protein bodies (PBs) during the late stage of seed maturation, followed by surrounding the PSV-derived membrane [6]. Whole flaxseed ranged from 18% to 30% of protein contents, which contained 70~85% of salt-soluble globulin and 15~30% of water-soluble albumin. The mass ratio and composition of albumin and globulin largely determined the techno-functionality of flaxseed proteins [7]. Flaxseed protein isolates (FPIs) from whole seed have been demonstrated to present better foaming capacity and stability than those from flaxseed protein concentrates due to their weaker electrostatic interaction between proteins, polysaccharides and phenolic compounds [8]. The adjustment of extraction substrates and recovery methods was effective to improve the techno-functionality of FPIs [9,10]. However, the preliminary tailoring of favorable emulsifying and foaming properties might be crucial for FPIs through screening the major cultivated flaxseed varieties.

Based on above, the current study aimed to investigate the varying composition structure and techo-functionality of FPIs obtained from the six flaxseed varieties, including Longya 13#, Baya 10#, Zhangya 2#, Neiya 9#, Dingya 25# and Ningya 21#, from the major producing areas in China. The screening of FPI with superior techno-functionality was definitely favorable for simplifying the downstream structural modifications of proteins and subsequent introduction in health food, and ultimately achieving the diversification and high-value utilization of flaxseed.

## 2. Materials and Methods

### 2.1. Chemicals and Materials

Flaxseed varieties were collected from the major planting areas in China from 2023 to 2024 through China Agriculture Research System for Specialty Oil Crops, including Longya 13#, Baya 10#, Zhangya 2#, Neiya 9#, Dingya 25# and Ningya 21#. Two biological replicates of each flaxseed variety were mixed in equal weights and stored at low temperature in sealed polyethylene bags prior to analysis. The Folin–Ciocalteu reagent, rutin and amino acid standards were purchased from Beijing Solarbio Sciences and Technology Co., Ltd. (Beijing, China). The 2,4,6-Tris(2-pyridyl)-striazine (TPTZ, 99%) and 2,2′-diphenyl-1-picrylhydrazyl radical (DPPH, 95%) were obtained from Shanghai Beyotime Biotechnology Co., Ltd. (Shanghai, China). The micro BCA Protein Assay Kit was bought from Beyotime Biotechnology Co., Ltd. (Shanghai, China). Other analytical-grade reagents were purchased from Sinopharm Chemical Reagent Co., Ltd. (Beijing, China).

### 2.2. Analysis of Microstructure of Protein Bodies from Different Flaxseed Varieties

According to the previous method, the in situ imaging of PBs in flaxseed was conducted using s transmission electronic microscope (TEM). TEM imaging was performed on a Hitachi H-7650 instrument (Tokyo, Japan) operated at 100 kV [11].

### 2.3. Preparation of FPIs from Different Flaxseed Varieties

The degummed flaxseed was freeze-dried, ground into fine powder using a coffee grinder, and defatted twice using hexane. The defatted flaxseed flours were dispersed in deionized water, adjusted to pH 8.5 using 0.5 M NaOH solution, stirred for 2 h at 25 °C and centrifuged at 8000× *g* for 20 min. Then, the supernatant was adjusted to pH value of 3.8 with 0.5 M HCl to precipitate protein, which was dispersed in deionized water and adjusted to pH value of 6.8. Then, the above protein dispersion was subjected to dialysis using a dialysis bag with molecular cut off weight of 6–8 kDa against deionized water for 48 h at 4 °C. The dialysis water was changed every 8 h, and the ratio of protein dispersion to deionized water was set to 1:50 (*v*/*v*). Then, the sample was freeze-dried to obtain FPIs [12].

### 2.4. Analysis of Surface Morphology, Protein Purity and Amino Acid Profiles of FPIs

The surface morphology of FPIs was observed by using high-resolution field emission scanning electron microscopy (FE-SEM) Regulus 8100 (Hitachi, Tokyo, Japan) at an accelerated voltage of 3.0 kV with magnification 10,000×. The purity of FPIs was determined using the automatic kjeldahl analyzer and calculated with a conversion factor of 6.25. Then, protein samples were hydrolyzed with 6 M HCl for 24 h at 110 °C, analyzed using a Biochrom 30 automatic amino acid analyzer (Biochrom Ltd., Cambridge, UK), and the results were expressed as mg/g protein (dry basis).

### 2.5. Analysis of Physicochemical Properties of FPIs

The mean particle sizes of FPI dispersions were evaluated through Dynamic Light Scattering (DLS), and zeta potential was evaluated through Electrophoretic Light Scattering (ELS) using a ZetaSizer Nano ZS 90 (Malvern Instruments Ltd., Malvern, UK). The protein solubility was calculated as the percentage of the soluble protein relative to total protein content in FPIs, which was dispersed in 50 mM PBS (pH 7.0) at a concentration of 1% (*m*/*v*).

### 2.6. Analysis of Composition Structure of FPIs

#### 2.6.1. Protein Subunit Profiles

Sodium Dodecyl Sulfate–Polyacrylamide Gel Electrophoresis (SDS–PAGE, 10%) was performed on FPIs (aqueous dispersion, 1.0 mg/mL) under reducing conditions in the presence of 5% β-mercaptoethanol. Then, gels were stained with Coomassie brilliant blue solution and imaged on a ChemiDoc XRS+ System (Bio-Rad, Hercules, CA, USA).

#### 2.6.2. Intrinsic Fluorescence Properties

The FPIs were diluted in deionized water (0.1 mg/mL) and analyzed for intrinsic fluorescence properties using an RF-7000 fluorescence spectrophotometer (Shimadzu, Kyoto, Japan). The excitation and scanning wavelength were set at 280 nm and 300–450 nm, respectively.

#### 2.6.3. Protein Secondary Structure

The dispersions of FPIs were diluted in 10 mM phosphate buffer (pH 7.0), and the protein concentration was verified at 0.1 mg/mL by absorbance at 280 nm. Far-UV circular dichroism (CD) spectra (190–260 nm, 1 nm bandwidth) were recorded using a Chirascan Plus spectropolarimeter (Applied Photophysics Ltd., Surrey, UK). Spectra were smoothed, baseline-corrected, and analyzed using the CDSSTR algorithm with the SP175 dataset to estimate the contents of α-helix, β-sheet, β-turn, and random coil.

### 2.7. Analysis of Emulsifying Properties of FPIs

#### 2.7.1. Emulsifying Activity and Stability

The emulsifying activity index (EAI) and emulsifying stability index (ESI) were calculated using the following equations [12]:

$$\mathrm{EAI}\;(m^{2}/g)\;=\;\frac{2\;\times \;2.303\;\times \;A_{0}\;\times \;\mathrm{DF}}{C\;\times \;\varphi \;\times \;10,000}$$

$$\mathrm{ESI}\;(\min)=\frac{A_{0}}{A_{0\;}-{\;A}_{10}}\;\times \;10$$

where DF is the dilution factor (100), C is the protein concentration (g/mL), φ is the oil volume fraction (0.25), and A_0_ and A_10_ are the absorbance values of coarse emulsions at 0 min and 10 min, respectively.

#### 2.7.2. Microstructure of Coarse Emulsions

The *in situ* microstructure of coarse lipid droplets and bulk continuous phase in coarse emulsions were visualized by using the high-resolution field emission scanning electron microscopy (FE-SEM) Regulus 8100 (Hitachi High-Tech, Tokyo, Japan) equipped with PP3010T Cyro-SEM Preparation System (including cryo-fracturation, gold coating) (Quorum Technologies Ltd., Ringmer, UK) [13].

#### 2.7.3. The Oil–Water Interfacial Activities

The oil–water interface tension (π) values of FPIs (1.0%, *m*/*v*) were measured by a K100 surface tensiometer (KRÜSS GmbH, Hamburg, Germany) using the Wilhelmy plate technique. The test was conducted over 3600 s, and the temperature was maintained at 25 °C throughout the duration of the test [14].

### 2.8. Analysis of Foaming Properties of FPIs

#### 2.8.1. Foaming Capacity and Stability

The foaming capacity (FC, %) and foaming stability (FS) were calculated using the following equations [9]:

FC (%) = V_0_/V_L_ × 100

FS (%) = V_1_/V_0_ × 100

where V_L_ is the volume of non-shearing protein dispersion, 15 mL; V_0_ is the volume of foams immediately after shearing; and V_1_ is the volume of foams 30 min after shearing.

#### 2.8.2. Microstructure of Crude Foams

The high-resolution field emission scanning electron microscope (FE-SEM) (Regulus 8100, Hitachi, Tokyo, Japan) equipped with a cryo preparation system (PP3010T, Quorum, UK) was used to study the micromorphology of coarse foams prepared using the dispersions of FPIs [15].

#### 2.8.3. The Air–Water Interfacial Activities

The air–water interfacial pressure (π) values of FPIs (1.0%, *m*/*v*) were determined by using the Wilhelmy plate technique. The test was conducted over 3600 s, and the temperature was maintained at 25 °C throughout the duration of the test [14].

### 2.9. Analysis of Phenolic Compounds and In Vitro Antioxidant Activities of FPIs

FPIs were extracted with methanol aqueous solution by vortex mixing and subsequent ultrasonic bath. Total phenolic acids and flavonoids of extracts were determined using the Folin–Ciocalteu and aluminum nitrate assays, respectively [16,17]. The results were expressed as mg gallic acid and rutin equivalents (mg/100 g) per 100 g sample (dry basis), respectively. The antioxidant activities of extracts were performed using DPPH and ferric reducing antioxidant power (FRAP) methods, and the values were expressed as mg ascorbic acid equivalents (mgAAE/100 g) per 100 g samples (dry basis), respectively.

### 2.10. Statistical Analysis

The data were presented as mean ± standard deviations (n = 3), and analyses were carried out with SPSS 24 for Windows (SPSS Inc., Chicago, IL, USA). One-Way ANOVA, followed by a Tukey test, was performed to analyze the significant differences between data (*p* < 0.05).

## 3. Results

### 3.1. The Microstructure of Flaxseed Protein Bodies from Different Varieties

**Figure 1:**
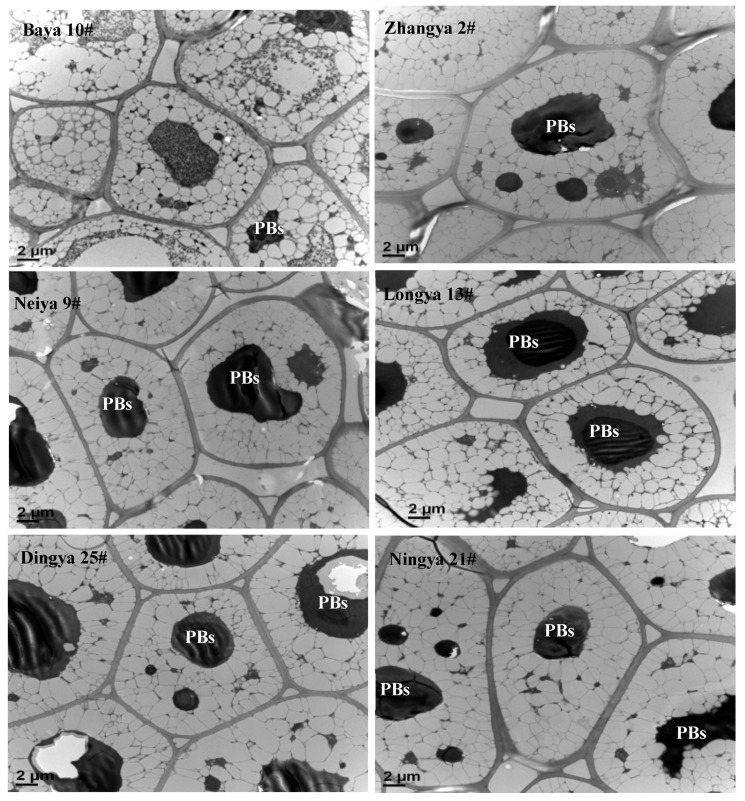
The occurrence forms of protein bodies from different flaxseed varieties. PBs: protein bodies.

As seen in Figure 1, the microscope observation of apparently intact PBs, the dynamic protein assemblies, manifested the densely staining cytoplasmic inclusions, which were located at the center of cotyledon cells in flaxseed. In particular, the densely packed storage protein deposits had distinct boundaries and varying diameters among different cotyledon cells in flaxseed. Meanwhile, OBs showed a round, oval and irregular shape, and were well distributed from the cell wall to the center in flaxseed. Notably, the membrane boundaries between large PBs and surrounding small OBs were clearly seen and were considered as the migration sites of lipid mobilization-related enzymes originally located at PBs. As for the variety specificity, PBs from the Dingya 25# and Ningya 21# flaxseed varieties had an oval shape, while those from the Baya 10#, Zhangya 2#, Neiya 9# and Longya 13# flaxseed varieties displayed an irregular shape. In particular, smaller amounts and sizes of PBs were observed for the Baya 10# flaxseed variety when compared with Zhangya 2#, Neiya 9#, Longya 13#, Dingya 25# and Ningya 21# flaxseed varieties. The PBs from the Dingya 25# and Longya 13# flaxseed varieties occupied a relatively higher proportion of the cotyledon cells in flaxseed. There was almost no other cell organ observed, except the nucleus with a relatively weak electron-dense nucleolus, further demonstrating that the intracellular space was filled with storage substances in cotyledon cells of flaxseed. However, the irregular shape, apparent coalescence and less dense appearance could have occurred in PBs subject to seed germination, which was also accompanied by a decrease in the number of OBs in flaxseed [18]. As previously reported, PBs were the main sites of storage protein particles in flaxseed, which began to form in the endosperm after several days of flowering. The disruption of PBs, storing several key enzymes, such as phospholipase A, lipase, and lipoxygenase, requisite for lipid mobilization, might explain their role in serving as an energy, carbon, and nitrogen source during seed germination [19]. Indeed, samples for ultrastructural observations were taken as nearly at the geometrical center of cotyledon sections of flaxseed. However, there were still evident appearance differences in the shape, size and discreteness of PBs that occurred among different flaxseed varieties. This might be related to the different spatial crowding effects contributed by the intracellular OBs with different particle sizes among the studied flaxseed varieties. And the formation, biochemistry and morphogenesis of PBs could have definitely been influenced by gene regulation and ultimately manifested as different protein contents and aqueous dispersion properties in different flaxseed varieties [20]. Understanding the microstructure of protein aggregations within PBs was particularly critical for formulating the isolation, composition structure and functionalities of proteins in flaxseed.

### 3.2. The Contents and Amino Acid Profiles of FPIs from Different Flaxseed Varieties

The nutritional functionality of food proteins is largely dependent on amino acid profiles and how closely the amino acid profile fits the ideal protein standard pattern [20]. According to the ideal amino acid composition standard proposed by WHO/FAO, the ratio of essential amino acids to total amino acids (E/T) should be higher than or close to 40%. As seen in Table 1, the E/T values of FPIs among different flaxseed varieties ranged from 38.97% to 41.46%, which is almost close to the standard value. The total amino acid contents of FPIs from different flaxseed varieties varied between 738.87 mg/g and 788.81 mg/g. Of these, FPIs from the Neiya 9# flaxseed variety had the most abundant total amino acid content, followed by those from Zhangya 2#, Dingya 25#, Longya 9#, and Ningya 21# flaxseed varieties. However, the lowest percentages of total amino acid contents were observed for FPIs from the Baya 10# flaxseed variety. Amino acids have been classified as nutritionally essential amino acids (EAAs) or nonessential amino acids (NEAAs) for the maintenance, growth, development, and survival of animals. The levels of EAAs for FPIs ranged from 287.93 mg/g to 316.02 mg/g among different flaxseed varieties, which was relatively lower than those reported in previous studies [21]. Among them, FPIs from the Ningya 21# flaxseed variety possessed the highest content of essential amino acids and highest E/T values, followed by those from the Zhangya 2#, Neiya 9# and Dingya 25# flaxseed varieties.

The hydrophobic and branched-chain amino acids provided structural stability through hydrophobic interactions, whereas negatively charged and sulfur-containing amino acids enhance function and stability via ionic interactions and disulfide bonds. The lowest and highest branched-chain, hydrophobic, negatively charged and sulfur-containing amino acids were observed for FPIs from the Baya 10# and Ningya 21# flaxseed varieties, respectively. FPIs from the Longya 13# flaxseed variety also possessed the lowest sulfur-containing amino acids, but had relatively higher branched-chain, hydrophobic, and negatively and positively charged amino acids. The comparative levels of positively charged amino acids were found for FPIs from the Baya 10#, Neiya 9#, Zhangya 2#, Dingya 25# and Longya 13# flaxseed varieties, which was significantly higher than those from the Ningya 21# flaxseed variety (*p* < 0.05). In particular, FPIs from Baya 10# and Ningya 21# flaxseed showed the lowest and highest branched-chain, hydrophobic, negatively charged and sulfur-containing amino acids, respectively. The different contents of hydrophobic and negatively and positively charged sulfur-containing amino acids could affect the conformation structure and physicochemical properties of FPIs, which actually further affected the interfacial behavior, namely the adsorption and rearrangement dynamics at the oil–water or air–water interfaces. Theoretically, the high contents of hydrophobic amino acids and sulfur-containing amino acids endowed the relatively ordered secondary structures, tunable structural properties, and acceptable interfacial behavior of FPIs [22]. In fact, besides the contents, the exposition degree of hydrophobic groups and negatively charged amino acids in the aqueous phase also largely contributed to the techno-functionality of FPIs.

The medicinal amino acids included Glu, Asp, Arg, Gly, Phe, Met, Leu, Tyr and Lys. The total medicinal amino acids in FPIs of different flaxseed varieties varied from 468.11 to 502.03 mg/g, with the highest and lowest contents observed for Zhangya 2# and Baya 10# flaxseed varieties, respectively. Among them, the highest leucine and isoleucine contents were obtained for FPIs from the Zhangya 2# and Dingya 25# flaxseed varieties, with values of 52.90 mg/g and 56.77 mg/g, respectively. By contrast, FPIs from the Ningya 21# flaxseed variety displayed the highest content of glutamic acid and aspartic acid, reaching 134.63 mg/g and 78.20 mg/g, respectively. The Arg content in FPIs from the Ningya 21# flaxseed variety was only 41.17 mg/g, which was much lower than those from other flaxseed varieties. However, the levels of Lysine in FPIs remained almost consistent among different flaxseed varieties. Studies have shown that medicinal amino acids are considered to be pivotal for organism life activities and for maintaining nitrogen balance. Dietary supplementation of several amino acids, such as Arg, Glu, Leu, etc., was involved in the modulation of gene expression, improvement of growth in the small intestine and skeletal muscle, and reduction in excessive body fat. Undoubtedly, the low Arg content in FPIs from the Ningya 21# flaxseed variety might reduce its nutritional quality for such processes as nitric oxide synthesis and immune regulation [23].

### 3.3. The Morphological Structure and Physicochemical Properties of FPIs from Different Flaxseed Varieties

**Figure 2:**
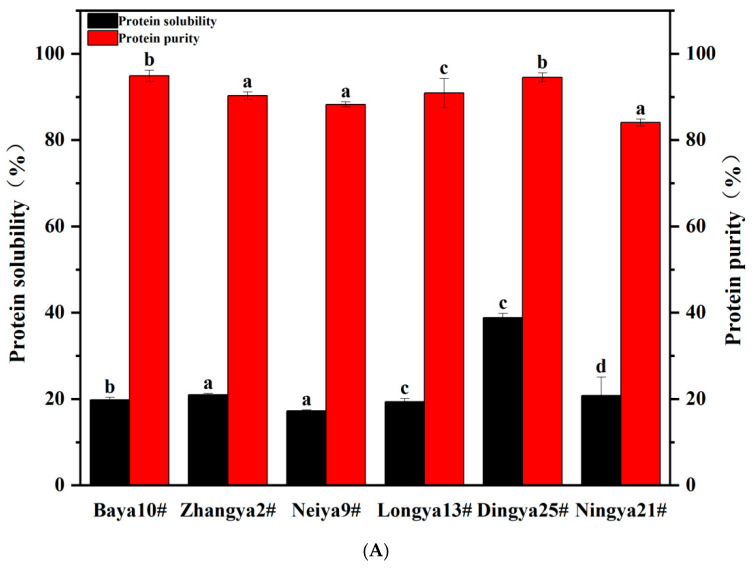

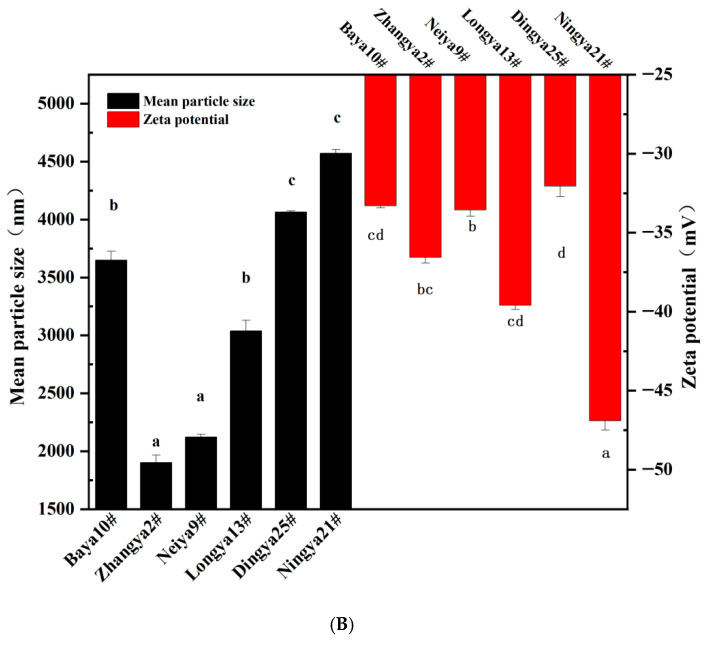
The physicochemical properties of FPIs from different flaxseed varieties. (**A**) The purity and solubility; (**B**) the average particle sizes and zeta potential values. Means with different letters are significantly different at the *p* < 0.05 level. FPIs, flaxseed protein isolates.

As previously reported, PBs in various seeds and nuts could be isolated by using the differential sedimentation method in a variety of media. In this study, FPIs were obtained from the defatted flours of whole flaxseed using the alkali solution acid precipitation method. As shown in Figure 2, the values of the protein purity of FPIs from different flaxseed varieties ranged from 84.07% to 94.90%. The highest protein purity was obtained for FPIs from Baya 10# and Dingya 25#, followed by Zhangya 2# and Neiya 9# flaxseed varieties, whereas the lowest protein purity was found in those from the Ningya 21# flaxseed variety. By contrast, the crude protein levels of FPIs prepared from dehulled and whole flaxseed reached 91% and 82%, respectively, when calculated using the same nitrogen-to-protein conversion factor [15,24]. In fact, except for the genetic background and environmental condition of flaxseed, the introduction of gum polysaccharides into FPIs based on their non-covalent interactions might have occurred during the aqueous extraction of whole defatted flours [25]. Flaxseed possesses a complex multi-layered coat, which consists of eight cellular tissue layers. In particular, the outermost cell layers contain gum polysaccharides with high intrinsic viscosity, which account for 8–10% of whole flaxseed and definitely reduce the recovery efficiency of proteins released from whole flaxseed [2].

Solubility had a significant impact on the techno-functionalities, predominantly the emulsifying and foaming properties of proteins. And solubility was also a commonly used indicator for measuring the denaturation and aggregation degree of proteins [26]. The protein solubility of FPIs from different flaxseed varieties ranged from 17.23% to 38.87%. The highest protein solubility was achieved for FPIs from the Dingya 25# flaxseed variety, and the lowest value was found for Neiya 9#, followed by the Longya 13#, Baya 10#, Ningya 21# and Zhangya 2# flaxseed varieties. As previously reported, albumin possesses smaller particle dimensions and superior protein solubility compared to globulin in flaxseed proteins due to its lower molecular weight and hydrophobicity [12]. In the current study, the potential negative relationship was further confirmed between the protein purity and solubility of FPIs in the studied flaxseed varieties. In fact, the residual gum polysaccharides could improve the solubility of FPIs by preventing aggregation and enhancing hydration through electrostatic interactions and steric stabilization [27].

Significant differences in the dispersion properties were observed for FPIs among different flaxseed varieties, with the mean particle sizes ranging from 1.9 μm to 4.5 μm, which is comparable to those found in native FPIs before high-hydrostatic-pressure treatment [24]. FPIs from the Ningya 21#, Dingya 25# and Baya 10# flaxseed varieties showed the highest mean particle sizes, with mean values of 4.10 μm. By contrast, FPIs from the Zhangya 2#, Neiya 9# and Longya 13# flaxseed varieties had the lowest mean particle sizes, with average values of 2.35 μm. The zeta potential values of FPIs varied from −33.30 to −46.90 mV, which was evidently higher than those in FPIs prepared from dehulled flaxseed [12]. The highest potential value was observed for FPIs from the Ningya 21# flaxseed variety, followed by Longya 13# and Zhangya 2# flaxseed varieties. By contrast, FPIs from Dingya 25#, Baya 10# and Neiya 9# flaxseed varieties revealed the lowest zeta potential values, with mean values of −32.98 mV. The interaction degree of globulin and albumin fractions in FPIs and the retention amounts of extrinsic components, such as negative-charged gum polysaccharides in FPIs, might jointly determine the dispersion behavior and charge densities of FPIs in the aqueous phase [28].

### 3.4. The Composition Structure of FPIs from Different Flaxseed Varieties

**Figure 3:**
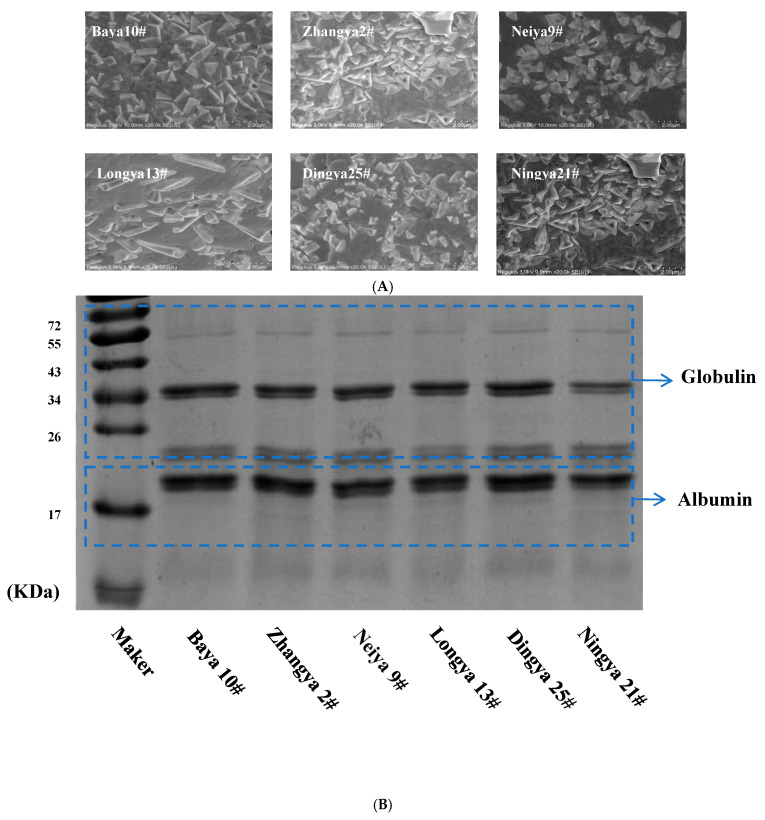

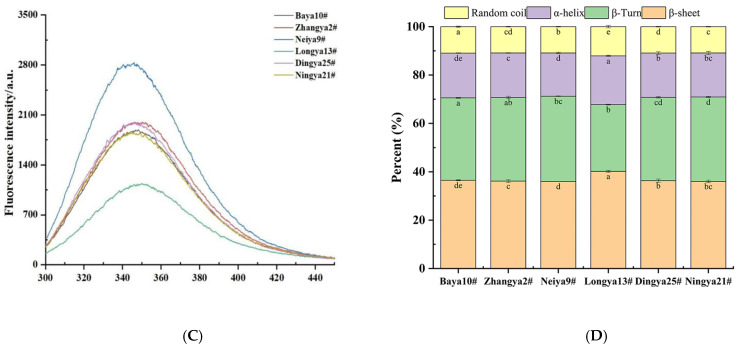
The composition structure of FPIs from different flaxseed varieties. (**A**) The micromorphology; (**B**) the subunit profiles; (**C**) the intrinsic fluorescence properties; (**D**) the secondary structure. Means with different letters are significantly different at the *p* < 0.05 level. FPIs, flaxseed protein isolates.

As depicted in Figure 3, after alkali extraction with isoelectric precipitation from whole defatted flaxseed flours, the protein aggregates exhibited a relatively small and irregular lamellar strip structure, which varied in size and shape among the studied flaxseed varieties. A high-intensity band was found for FPIs at 17~26 kDa and approximately 34 kDa, followed by extremely light bands at approximately 55 kDa. The globulin was composed of several polypeptides in the 10~50 kDa range, while albumin mainly consisted of the 10 kDa polypeptide accompanied by a minor content of 40 kDa [8,29]. No evident differences in the band profiles and intensities were observed for FPIs among the studied flaxseed varieties. This indicated that the composition structure specificity, namely the interaction degree between albumin and globulin, could be manifested for FPIs from different flaxseed varieties.

The aromatic amino acid residues—including tyrosine, tryptophan and phenylalanine—in protein molecules could emit intrinsic fluorescence signals in neutral aqueous solution, revealing the structural conformation changes in proteins upon various processing [30]. FPIs presented the maximum fluorescence emission spectrum (λ_max_) at 350 nm. However, FPIs recovered from dehulled whole flaxseed presented a λ_max_ at 331.6 nm [31]. The introduction of gum polysaccharides undoubtedly affected the spatial conformation of FPIs due to the non-covalent interactions between them [22]. Among them, the maximal value of maximum fluorescence intensity (FI_max_) was observed for FPIs from the Neiya 9# flaxseed variety. By contrast, an evident quenching of FI_max_ and a 350.20 nm red-shift in λ_max_ were concurrently detected for FPIs from the Baya 10#, Zhangya 2#, Dingya 25# and Ningya 21# flaxseed varieties. The lowest FI_max_ at λ_max_ was exhibited for FPIs from the Longya 13# flaxseed variety. The relatively mild differences in the contents of aromatic amino acids acting as fluorophores, as well as the embedding degree in protein hydrophobic cores due to their different spatial conformality, could explain the variety specificity of fluorescence intensity for FPIs among the studied flaxseed varieties [28].

FPIs from the Longya 13# flaxseed variety contained 20.14% α-helix, 40.18% β-sheet, 27.60% β-turns and 12.08% random coil, respectively. By contrast, obviously lower α-helix and β-sheet levels but a higher β-turn content were determined for FPIs from Neiya 9#, Baya 10#, Zhangya 2#, Dingya 25# and Ningya 21# flaxseed varieties. The contents of random coil of FPIs displayed statistically non-significant differences among Longya 13# and Ningya 21# flaxseed varieties. FPIs in aqueous phase displayed a relatively ordered structure due to their high β-sheet and lower random coil contents [32]. FPIs also contained a relatively low content of α-helix and a high content of β-sheet, further confirming the relatively ordered structure [33].

These indicated that the polypeptide chains of FPIs from the Longya 13# flaxseed variety tended to form more stable interfacial films with enhanced emulsifying and foaming stability, but had a limited ability to rapidly adsorb and unfold at gas–water and oil–water interfaces. In contrast, FPIs from other flaxseed varieties, particularly for Neiya 9#, tended to form a spatial conformation with higher molecular flexibility, which improved emulsifying and foaming capacity due to higher proportions of random coils and β-turns [34].

### 3.5. The Emulsifying Properties of FPIs from Different Flaxseed Varieties

**Figure 4:**
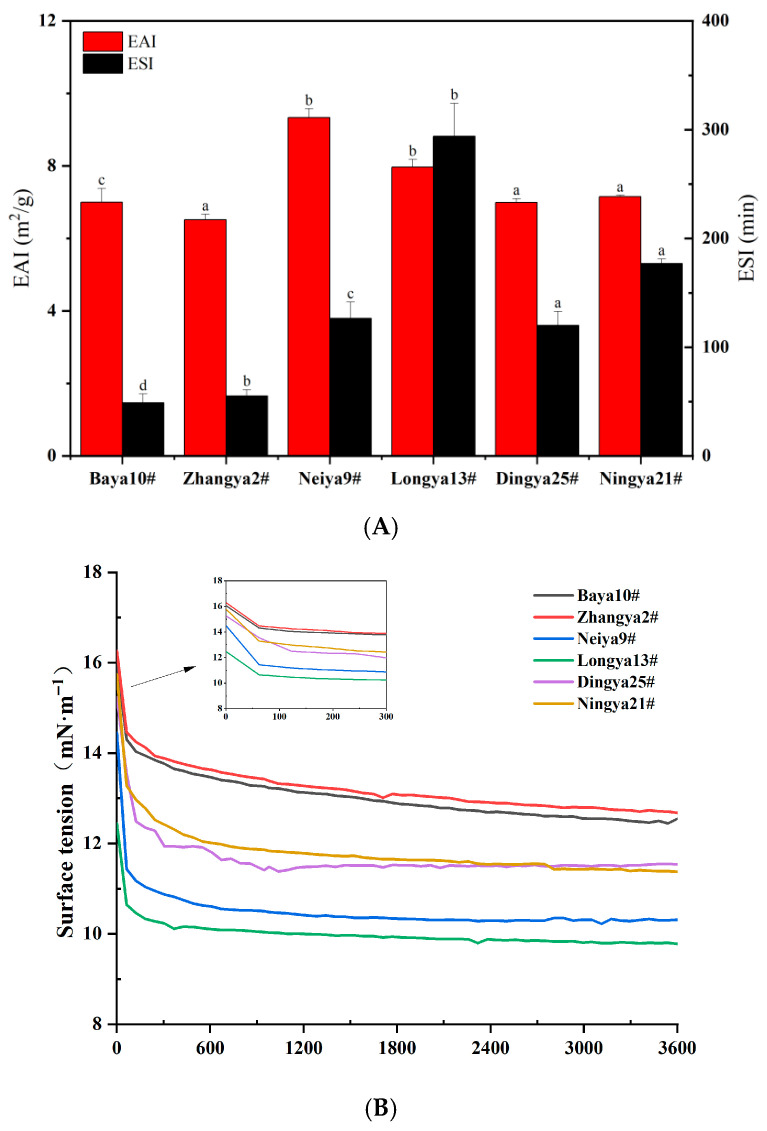

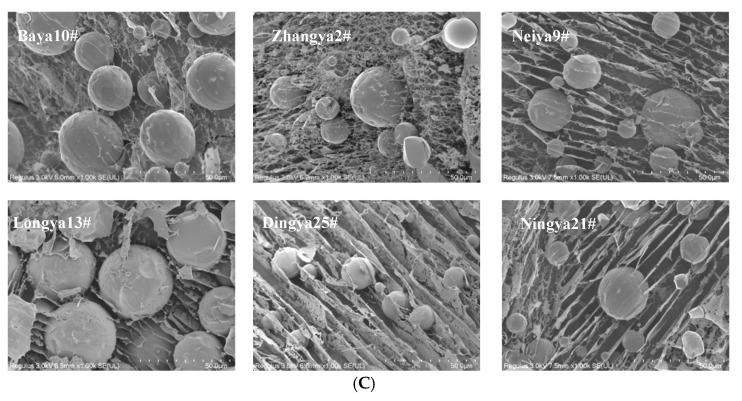
The emulsifying properties of FPIs from different flaxseed varieties. (**A**) Emulsifying capacity and stability; (**B**) the oil–water interface activity; (**C**) microstructure of emulsions prepared by FPIs. Means with different letters are significantly different at *p* < 0.05 level. FPIs, flaxseed protein isolates.

The emulsification activity index (EAI) and emulsification stability index (ESI) are usually used to evaluate the emulsification properties of proteins [35]. As shown in Figure 4, the EAI values of FPIs from different flaxseed varieties ranged from 6.52 to 9.33 m^2^/g. Among them, the highest EAI value was observed for FPIs from Neiya 9#, followed by the Longya 13# flaxseed variety. FPIs from the Zhangya 2# flaxseed variety possessed the lowest EAI value, which accounted for 69.98% of that of the Neiya 9# flaxseed variety (*p* < 0.05). The ESI values of FPIs among different flaxseed varieties ranged from 48.99 to 293.91 min. Of these, FPIs from the Longya 13# flaxseed variety had the highest ESI value, followed by FPIs from Ningya 21#, Neiya 9# and Dingya 25# flaxseed varieties. By contrast, FPIs from the Baya 10# flaxseed variety exhibited the lowest ESI value, which just accounted for 16.67% of that of the Longya 13# flaxseed variety (*p* < 0.05). The emulsification capacities of FPIs could be highly related to the amounts of hydrophobic amino acid residues on the surface of protein molecules, but not just to the total amounts of hydrophilic groups of proteins. Our previous finding indicated that the albumin and globulin fractions contributed to the emulsifying activity and stability of FPIs, respectively [12]. Thus, an appropriate composition structure, namely the interaction degree between albumin and globulin, might explain the relatively superior emulsifying properties of FPIs from the Longya 13# flaxseed variety when compared to those from Baya 10#, Zhangya 2#, Neiya 9#, Dingya 25# and Ningya 21# flaxseed varieties. Moreover, the relatively high zeta potential values of FPIs from Ningya 21#, followed by the Longya 13# flaxseed variety, were observed due to the abundant negatively charged amino acid residues. These indicated that the greater electrostatic repulsion between their protein molecules could inhibit the aggregation of emulsion droplets and thereby improve the emulsification stability of FPIs.

As an amphiphilic biomacromolecule, protein can achieve desirable adsorption on the oil–water interface during the preparation of lipid droplets. The time-dependent changes in interfacial tension values could reflect the instantaneous migration and adsorption capacity of protein molecules towards the oil–water interfaces [36]. The initial surface tension (π) value of FPIs from different flaxseed varieties in aqueous dispersion ranged from 12.44 mN·m^−1^ to 16.25 mN·m^−1^. FPIs from Longya 13# and Neiya 9# flaxseed varieties manifested the lowest π values, with the equilibrium time extended to 3600 s, followed by those from the Dingya 25# and Ningya 21# flaxseed varieties, whereas those from the Baya 10# and Zhangya 2# flaxseed varieties presented a minimum interfacial dropping trend over time. The microstructure of lipid droplets and the bulk continuous phase in the coarse emulsion prepared using FPIs was further explored through cryo-SEM imaging. FPIs from the Longya 13# flaxseed variety produced lipid droplets with uniform particle sizes (20~40 μm) but small amounts of lamellar network structures in the bulk aqueous phase, which further confirmed the desirable emulsifying capacity. Thus, more substantial conformation reorganization and greater adsorption capacity could be elucidated from the efficient migration and interface adsorption of FPIs from the Longya 13# flaxseed variety. By contrast, lipid droplets with narrow ranges of particle sizes (15~40 μm) were also observed for the emulsion prepared using FPIs from the Baya 10# flaxseed variety. Notably, the hollow filament connection, but not the lamellar network structure in the bulk aqueous phase, demonstrated the relatively inferior emulsifying stability of FPIs from the Baya 10# flaxseed variety. However, FPIs from Neiya 9#, Dingya 25# and Ningya 21# flaxseed varieties produced sparse lipid droplets with heterogeneous dimensions, which were attached on the compact single-, double- or multi-layer lamellar gel network structure in the bulk aqueous phase. Extremely heterogeneous lipid droplets (5~40 μm) were also found for the emulsion prepared using FPIs from the Zhangya 2# flaxseed variety, but these were dispersed in the loose reticular structure formed by filamentous substances in the bulk aqueous phase. As reported in our previous study, lipid droplets with loose/porous interfaces were produced by albumin, whereas interlayer anchoring but no direct interface coating was observed for lipid droplets constructed by the globulin fraction of FPIs [12].

### 3.6. The Foaming Properties of FPIs from Different Flaxseed Varieties

**Figure 5:**
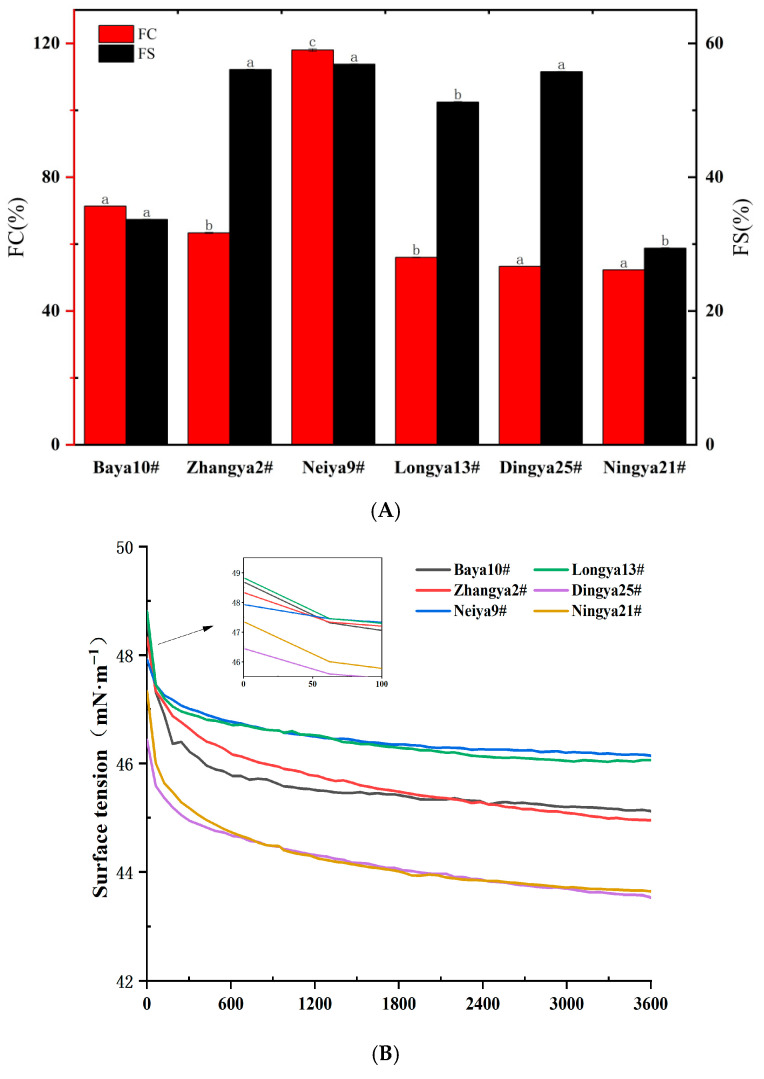

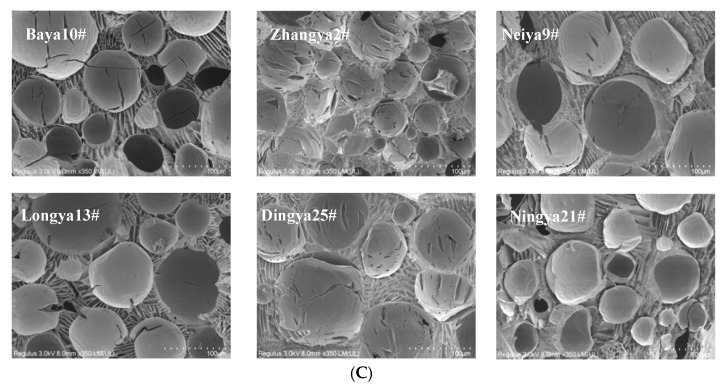
The foaming properties of FPIs from different flaxseed varieties. (**A**) Foaming capacity and stability; (**B**) gas–water interface activity; (**C**) microstructure of foams prepared using FPIs. Means with different letters are significantly different at the *p* < 0.05 level. FPIs, flaxseed protein isolates.

The foaming capacity (FC) and foaming stability (FS) were studied to evaluate the foaming properties of proteins [35]. As shown in Figure 5, the values of FC for FPIs from different flaxseed varieties ranged from 52.3% to 118.00%. Of these, the highest value of FC was observed for FPIs from Neiya 9#, followed by those from Baya 10# and Zhangya 2#, followed by the Longya 13#, Dingya 25# and Ningya 21# flaxseed varieties. By contrast, the lowest FC was found for the Ningya 21# flaxseed variety, reaching only 44.3% of that of the Neiya 9# flaxseed variety (*p* < 0.05). The values of FS for FPIs from different flaxseed varieties ranged from 29.38% to 56.88%. Of these, FPIs from the Dingya 9# flaxseed variety had the highest FS value, followed by the Zhangya 2# flaxseed variety. By contrast, FPIs from the Ningya 21# flaxseed variety had the lowest FS value, only accounting for 51.65% of the Neiya 9# flaxseed variety (*p* < 0.05). Thus, desirable foaming capacity and stability were screened for FPIs from Neiya 9# flaxseed.

Protein molecules can diffuse, and some protein subunits selectively adsorb, at gas–water interfaces, and they are then subjected to a conformational arrangement within the interface. The lower the interfacial tension at gas–water interfaces, the stronger the adsorption capacity of protein molecules. The initial surface tension (π) value of FPIs from different flaxseed varieties in aqueous dispersions ranged from 46.44 mN·m^−1^ to 48.81 mN·m^−1^. Of these, FPIs from the Dingya 25# flaxseed variety manifested the lowest initial π value, followed by those from Ningya 21#, Neiya 9# and Zhangya 2# flaxseed varieties. However, FPIs from the Baya 10# and Longya 13# flaxseed varieties exhibited the highest initial π values. Notably, the π values of FPIs from the Ningya 21# flaxseed variety dropped more sharply, which was comparable to those from the Dingya 25# flaxseed variety, with the equilibrium time extended from 600 s to 3600 s. The π values of FPIs from the Baya 10# flaxseed variety showed a maximal interfacial dropping trend, and were almost equal to those of FPIs from the Zhangya 2# flaxseed variety when the equilibrium time reached 1800 s. FPIs from the Neiya 9# flaxseed variety had relatively lower initial π values, but presented a minimum interfacial dropping range, which was comparable to that of FPIs from the Longya 13# flaxseed variety at an early equilibrium time.

The microstructure of coarse foams prepared using FPIs was further explored through cryo-SEM imaging. The foam freshly prepared from FPIs was perfectly round with a compact film, which was anchored into the gel network structure. Among them, the foams prepared using FPIs from Baya 10# and Longya 13# flaxseed varieties were distributed in a relatively narrow range (40~100 μm). By contrast, FPIs from Neiya 9# and Dingya 25# flaxseed varieties produced a relatively sparse foam distribution, with large but relatively uniform sizes (40~100 μm), whereas those from Zhangya 2# and Ningya 21# flaxseed varieties displayed foams with small and uniform diameters (20~70 μm). The gel network structure formed by filamentous substances was observed for the bulk aqueous phase of foams prepared using FPIs from the Zhangya 2# flaxseed variety, but a compact and lamellar gel network structure was found for those from Baya 10#, Longya 13#, Neiya 9#, Ningya 21# and Dingya 25# flaxseed varieties. Interfacial films with a relatively cracked structure were observed for FPIs from Baya10 #, Zhangya 2# and Dingya 25# flaxseed varieties, which could inevitably cause rapid drainage but no coalescence or collapse. By contrast, the tight contacts between adjacent films observed for FPIs from the Ningya 21# flaxseed variety could inevitably lead to a rapid coalescence or collapse after formation, suggesting a relatively inferior foaming stability. The interfacial migration and adsorption dynamics of albumin and globulin fractions in FPIs and the corresponding intermolecular interactions between them at air/water interfaces could maintain the interfacial compactness of foams against gas spillage. And the concurrent formation of a lamellar gel network in the bulk aqueous phase could also restrain the coalescence and collapse of adjacent foams created using FPIs [29].

### 3.7. The Retention of Phenolic Compounds and Antioxidant Activity of FPIs from Different Flaxseed Varieties

**Figure 6:**
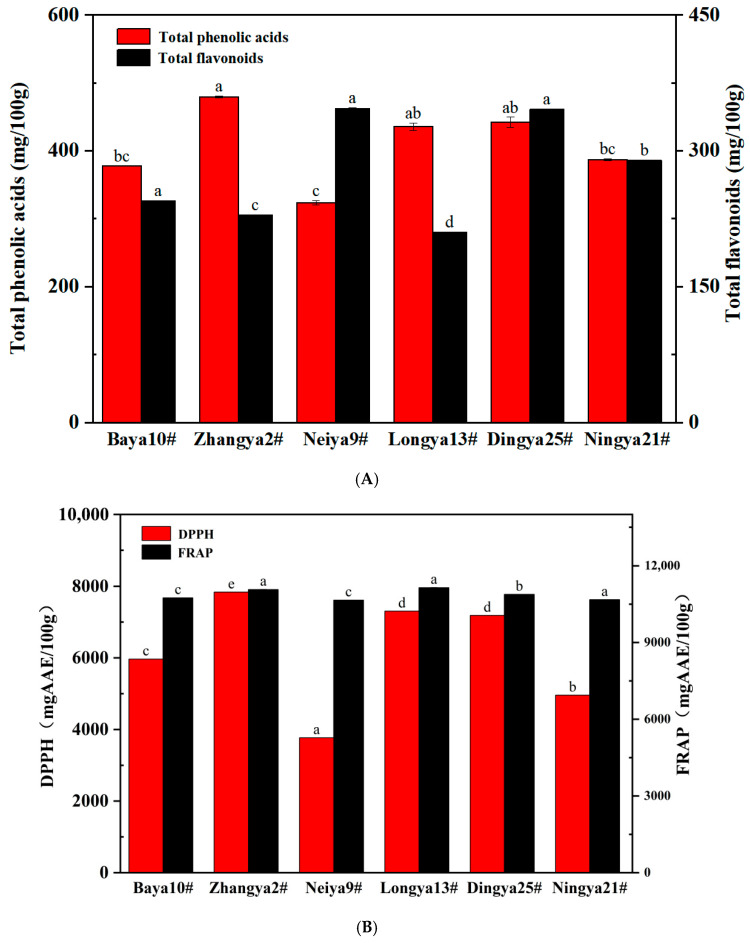
The retention of phenolic compounds and in vitro antioxidant activities of FPIs from different flaxseed varieties. (**A**) Phenolic compounds; (**B**) DPPH and FRAP values. Means with different letters are significantly different at the *p* < 0.05 level. FPIs, flaxseed protein isolates.

The retention of phenolic compounds was identified in the specific non-covalent interactions of FPIs during the extraction process [37]. Theoretically, the phenolic compounds were preferably accumulated into albumin when compared with those in the globulin fraction of FPIs [12]. As seen in Figure 6, the contents of total phenolic acids and flavonoids in FPIs from different flaxseed varieties ranged from 323 to 478 mg/100 g and 210 to 347 mg/100 g, respectively. Of these, the highest content of total phenolic acids was observed for FPIs from the Zhangya 2# flaxseed variety, followed by the Longya 13# and Dingya 25# flaxseed varieties, with an average of 452 mg/100 g. By contrast, the lowest contents of total phenolic acids were found in those from Ningya 21#, Baya10# and Neiya 9# flaxseed varieties, with mean values of 363 mg/100 g. FPIs from Neiya 9# and Dingya 25# flaxseed varieties showed the highest contents of total flavonoids, followed by the Ningya 21# flaxseed variety. However, FPIs from Baya 10#, Zhangya 2# and Longya 13# flaxseed varieties revealed the lowest levels of total flavonoids. The DPPH free radical scavenging activities of FPIs from different flaxseed varieties ranged from 3768 to 7836 mg AAE/100 g. FPIs from the Zhangya 2# flaxseed variety possessed the highest DPPH values, followed by those from Longya 13#, Dingya 25# and Baya 10# flaxseed varieties, whereas the lowest values were observed for Ningya 21# and Neiya 9# flaxseed varieties. The FRAP values of FPIs from different flaxseed varieties varied from 10,657 to 11,143 mg AAE/100 g, revealing no evident flaxseed variety specificity. An intimate positive relationship was particularly obtained between DPPH values and contents of total phenolic acids (R^2^ = 0.916), but not flavonoids, in FPIs from the studied flaxseed varieties. In fact, the retention of phenolic compounds definitely enhanced the antioxidant activities, but also partially weakened the interfacial properties, which essentially governed the formation and aging of lipid droplets and foams produced by FPIs [32,37]. FPIs prepared from dehulled and defatted flaxseed flours and then subjected to dialysis purification possessed a relatively lower retention of phenolic compounds [12]. In fact, flaxseed hulls contained relatively abundant phenolic compounds, mainly for lignan oligomers in the secondary wall of sclerite cells [38]. Therefore, the different flaxseed varieties, extraction substrates and recovery methods jointly affected the retention amounts of phenolic compounds in protein fractions prepared from flaxseed. The retention of phenolic compounds and non-covalent interactions with protein fractions might induce changes in the spatial conformation of FPIs by competing with intramolecular hydrogen bonding and hydrophobic interactions. The appropriate perturbations could improve the protein flexibility and surface hydrophobicity of FPIs due to partial subunit unfolding, which was favorable for balancing the interfacial adsorption and stabilization at the oil–water or air–water interface.

## 4. Conclusions

In summary, flaxseed variety significantly influenced the composition structure and selected techno-functionality of the resulting protein isolates. Variations in amino acid profiles, secondary structure, subunit distribution and spatial conformations of FPIs led to distinct interfacial behaviors, with specific varieties excelling in emulsification or foaming properties. Additionally, variety-dependent differences in the retention of co-extracted phenolic compounds enhanced in vitro antioxidant activities of FPIs, which was crucial for improving its interfacial functionality as a surface-active component. Thus, variety screening could serve as an effective initial strategy to improve the selected techno-functionality of FPIs by tailoring the intrinsic composition structure of FPIs and retention of phenolic compounds. In particular, Longya 13# and Neiya 9# flaxseed varieties were optimal materials for obtaining protein isolates with tailoring molecular rigidity and unfolding behavior. These specific intrinsic structural properties and the phenolic compound retention allowed us to balance the interfacial stability and capacities of FPIs when acting as emulsifying and foaming agents, respectively.

## Figures and Tables

**Table 1:** Amino acid profiles of FPIs from different flaxseed varieties.

Amino Acids (mg/g Protein)	Baya 10#	Zhangya 2#	Neiya 9#	Longya 13#	Dingya 25#	Ningya 21#
Essential amino acids (EAAs)						
Threonine (Thr)	27.28	30.56	29.38	28.48	29.16	28.44
Valine (Val)	44.52	41.11	45.08	46.47	43.42	46.68
Methionine (Met)	34.10	41.10	41.27	35.59	39.10	45.37
Isoleucine (lle)	47.35	52.00	53.50	49.42	56.77	51.530
Leucine (Leu)	48.78	52.90	52.53	50.91	51.53	52.21
phenylalanine (Phe)	40.14	42.09	42.65	41.89	42.13	44.48
Histidine (His)	20.58	21.42	22.18	21.48	21.27	20.91
Lysine (Lys)	25.20	25.69	26.01	26.30	24.45	26.40
Nonessential amino acids (NEAAs)						
Alanine (Ala)	31.87	33.55	35.05	33.27	33.49	32.19
Glycine (Gly)	34.84	36.93	38.71	36.36	36.23	38.36
Proline (Pro)	60.15	53.46	60.90	62.79	54.19	50.340
Serine (Ser)	35.08	37.99	37.68	36.61	36.74	37.05
Glutamic acid (Glu)	120.15	128.87	127.03	125.41	128.02	134.63
Aspartic acid (Asp)	69.50	74.10	74.35	72.55	75.98	78.20
Arginine (Arg)	71.81	75.71	73.08	74.95	73.29	41.17
Cystine (Cys)	3.94	4.42	4.12	4.12	5.42	7.60
Tyrosine (Tyr)	23.59	24.64	25.28	24.63	24.29	26.75
Total amino acids	738.87	776.55	788.81	771.22	775.49	762.31
Medicinal amino acids	468.11	502.03	500.91	488.59	495.02	487.58
E/T	0.39	0.40	0.40	0.39	0.40	0.41
BCAA	140.65	146.01	151.11	146.8	151.72	150.42
HAA	270.35	287.39	295.36	282.18	290.73	299.20
NCAA	189.65	202.97	201.38	197.96	204.00	212.84
PCAA	117.59	122.82	121.27	122.73	119.01	88.49
SCAA	38.04	45.52	45.39	39.71	44.52	52.97

Note: E/T: essential amino acids/total amino acids; medicinal amino acids: sums of Glu, Asp, Arg, Gly, Phe, Met, Leu, Tyr and Lys; BCAAs: branched-chain amino acids, including Val, Leu and Ile; HAAs: hydrophobic amino acids, including Ala, Val, Leu, Ile, Phe, Pro, Met, Gly and Tyr; NCAAs: negatively charged amino acids, including Asp and Glu; PCAAs: positively charged amino acids, including Lys, Arg and His; SCAAs: sulfur-containing amino acids, including Met and Cys. FPIs, flaxseed protein isolates.

## Data Availability

The original contributions presented in the study are included in the article, further inquiries can be directed to the corresponding authors.
